# Miltefosine and Antimonial Drug Susceptibility of *Leishmania Viannia* Species and Populations in Regions of High Transmission in Colombia

**DOI:** 10.1371/journal.pntd.0002871

**Published:** 2014-05-22

**Authors:** Olga Lucía Fernández, Yira Diaz-Toro, Clemencia Ovalle, Liliana Valderrama, Sandra Muvdi, Isabel Rodríguez, María Adelaida Gomez, Nancy Gore Saravia

**Affiliations:** 1 Centro Internacional de Entrenamiento e Investigaciones Médicas (CIDEIM), Cali, Colombia; 2 Centro Dermatológico Federico Lleras Acosta (CDFLLA), Bogotá, Colombia; New York University, United States of America

## Abstract

**Background:**

Pentavalent antimonials have been the first line treatment for dermal leishmaniasis in Colombia for over 30 years. Miltefosine is administered as second line treatment since 2005. The susceptibility of circulating populations of *Leishmania* to these drugs is unknown despite clinical evidence supporting the emergence of resistance.

**Methodology/Principal Findings:**

*In vitro* susceptibility was determined for intracellular amastigotes of 245 clinical strains of the most prevalent *Leishmania Viannia* species in Colombia to miltefosine (HePC) and/or meglumine antimoniate (Sb^V^); 163, (80%) were evaluated for both drugs. Additionally, susceptibility to Sb^V^ was examined in two cohorts of 85 *L. V. panamensis* strains isolated between 1980–1989 and 2000–2009 in the municipality of Tumaco. Susceptibility to each drug differed among strains of the same species and between species. Whereas 68% of *L. V. braziliensis* strains presented *in vitro* resistance to HePC, 69% were sensitive to Sb^V^. Resistance to HePC and Sb^V^ occurred respectively, in 20% y 21% of *L. panamensis* strains. Only 3% of *L. V. guyanensis* were resistant to HePC, and none to Sb^V^. Drug susceptibility differed between geographic regions and time periods. Subpopulations having disparate susceptibility to Sb^V^ were discerned among *L. V. panamensis* strains isolated during 1980–1990 in Tumaco where resistant strains belonged to zymodeme 2.3, and sensitive strains to zymodeme 2.2.

**Conclusions/Significance:**

Large scale evaluation of clinical strains of *Leishmania Viannia* species demonstrated species, population, geographic, and epidemiologic differences in susceptibility to meglumine antimoniate and miltefosine, and provided baseline information for monitoring susceptibility to these drugs. Sensitive and resistant clinical strains within each species, and zymodeme as a proxy marker of antimony susceptibility for *L. V. panamensis,* will be useful in deciphering factors involved in susceptibility and the distribution of sensitive and resistant populations.

## Author Summary

Treatment of dermal leishmaniasis is unsuccessful in an important proportion of cases and evidence of loss of susceptibility to antimonial drugs and miltefosine has been demonstrated in some cases of treatment failure with these medications. Despite the variability in the clinical outcome of treatment, little is known about the susceptibility of the different species and diverse populations of these species circulating in areas of high transmission. Based on 402 strains isolated from patients, the susceptibility of the *Leishmania* species most frequently causing dermal leishmaniasis in Colombia was determined to first and second line medications commonly used in Latin America. The results showed that susceptibility to each drug varied among the species and populations of each species, geographically between regions east and west of the Andes mountain range, during different time periods over 30 years, and within different epidemiological circumstances. The findings provide a comprehensive picture of drug susceptibility of dermal leishmaniasis in Colombia and baseline information for monitoring the emergence of drug resistance.

## Introduction

Pentavalent antimonial drugs, (sodium stibogluconate and meglumine antimoniate) have been the first-line monotherapeutic treatment for dermal leishmaniasis in Central and South America for decades. Parenteral administration, volumes of drug requiring divided doses in adults, frequent and potentially severe adverse effects, and the logistical challenges of accessing medical supervision of therapy contribute to interruption and abandonment of treatment, clinical failure, and loss of susceptibility [1], [2], [3]. Miltefosine (hexadecylphosphocholine), an oral drug originally developed for treatment of cancer, was approved in 2005 for the treatment of cutaneous leishmaniasis in adults and children in Colombia, and is administered as second line treatment in patients having contraindications or who fail to respond to antimonial drugs. Miltefosine has demonstrated efficacy comparable to pentavalent antimonials against infection caused by *Leishmania Viannia panamensis* in Colombia [4], [5], *L. V. guyanensis* [6] and *L. V. braziliensis* in Brazil [7] and *L. V. braziliensis* in Bolivia [8]. Nevertheless, treatment failures and relapses have been observed for both treatments in these and other randomized controlled clinical trials [5], [9].

Multiple factors contribute to the outcome of treatment, including those of the host (immune status, pharmacokinetics, pharmacogenetics, drug metabolism, adherence to treatment), pharmacological properties of the drug (formulation and pharmacodynamics) and parasite characteristics (biochemical and molecular differences among species and strains) [4], [10]. Evidence for the emergence drug resistance to antimonial drugs [11], [12] and miltefosine [13], [14] has been reported for visceral and cutaneous leishmaniasis. Although the role of drug susceptibility in treatment failure has been difficult to establish because therapeutic response is multifactorial, prospective analyses of strains isolated prior to treatment and at failure have demonstrated that loss of susceptibility to antimony and to miltefosine can contribute to treatment failure in dermal leishmaniasis [12], [13].

Reports based on clinical response to different drugs in diverse geographical settings indicate wide variation in effectiveness [4], [6], [7], [8], [15]. In Colombia, where *L. V. panamensis* is the predominant species isolated from civilian patient populations [16], [17], [18], 91% of patients responded to miltefosine treatment, whereas only 53% of patients responded in Guatemala, where *L. mexicana* and *L. V. braziliensis* are common [4]. Cutaneous leishmaniasis caused by *L. V. braziliensis* in Guatemala was highly responsive to sodium stibogluconate [19] whereas disease caused by *L. V. guyanensis* was poorly responsive to treatment with meglumine antimoniate in Brazil [15] but highly responsive in Peru [20]. Although some of these reports are not based on randomized, controlled clinical trials, they portray the therapeutic response in endemic settings.

*In vitro* evidence of inter species differences in the susceptibility of *Leishmania* to antileishmanial drugs has also been reported based on small numbers of strains of diverse species isolated from patients with visceral as well as cutaneous disease [21], [22]. Technical constraints in evaluating EC_50_ based on intracellular survival of clinical strains of *Leishmania* have limited routine or large scale assessment of drug susceptibility, and prevented the understanding of drug susceptibility in the context of prevention and control.

This large scale study of susceptibility to drugs in use in Colombia documented significant differences in susceptibility to miltefosine and meglumine antimoniate among strains of the same species and between species of the Viannia subgenus, as well as geographic variation in drug susceptibility of populations of these species. Additionally, this analysis demonstrated for the first time, intrinsic differences in the susceptibility to meglumine antimoniate between phenotypically distinct populations of *L. V. panamensis*.

## Materials and Methods

### Study design

This study sought to determine the susceptibility of the most prevalent species of Leishmania in Colombia to antileishmanial drugs currently in use, and to explore possible geographic variations in drug susceptibility of the corresponding parasite populations. The in vitro susceptibility of clinical strains of L. V. panamensis, L. V. braziliensis and L. V. guyanensis isolated at diagnosis from patients who acquired infection in foci of transmission in the Pacific, Andean, Orinoquía and Amazon regions of Colombia was evaluated. Since treatment with pentavalent antimonials has been the standard of care in Colombia since 1980, we also examined whether the susceptibility to SbV of L. V. panamensis, the most prevalent species among civilian patients, changed over the 10 year periods from 1980–1989 and 2000–2009 in two foci of transmission in the municipality of Tumaco on the Pacific coast of Colombia. Clinical and community based studies conducted in Tumaco since 1980 have contributed to the collection and characterization of clinical strains based on isoenzyme polymorphism profiles (zymodeme) [16]. These resources and demographic and epidemiologic variables (residence within focus of transmission, age, sex and occupation) of the corresponding patients in these endemic foci were exploited to examine epidemiologic associations.

### Strains

All *Leishmania Viannia* strains were isolated at diagnosis from patients with cutaneous lesions by medical personnel in two national reference centers for leishmaniasis, and cryopreserved in liquid nitrogen in the biobanks of these centers: *Centro Internacional de Entrenamiento e Investigaciones Médicas*, *CIDEIM*, Cali, and the *Centro Dermatológico Federico Lleras Acosta, CDFLLA*, Bogotá, Colombia. *In vitro* susceptibility was evaluated within four passages from isolation. Species identification was achieved by isoenzyme electrophoresis or indirect immunofluorescence using species-specific monoclonal antibodies as described elsewhere [16], [17]. Prior to conducting drug susceptibility assays, the species identity was re-confirmed for approximately 70% of the strains included in this study.

The proportion of strains of each species that was evaluated was based on the relative prevalence of the species in each of the regions of high transmission; 245 strains were evaluated: 163 for both drugs, plus 41 for SbV only and 41 for HePC only. The number of strains of each species that were evaluated for susceptibility to SbV and/or HePC, is summarized in Table 1. Additionally, in order to characterize the profile of susceptibility to SbV in an area of high transmission and long-term use of this drug, we evaluated 170 clinical strains of L. V. panamensis isolated from patients residing in transmission foci along the Rosario and Mira rivers; 85 of the 170 strains were isolated between 1980 and 1989 following the initiation of the national leishmaniasis control program, and 85 between 2000 and 2009; 13 of the latter strains were included in the sample of L panamensis evaluated for susceptibility to SbV and HePC from the Pacific Coast region.

**Table 1:** Species distribution of *L. Viannia* strains from regions of high prevalence evaluated *in vitro* for drug susceptibility^a^.

	Number of strains evaluated per species (%)			Total Per Drug
	*L. V. panamensis*	*L. V. braziliensis*	*L. V. guyanensis*	
**Sb^V^**	117 (57)	58 *(*28)	29 (15)	**204**
**HePC**	107 (52)	63 (31)	34 (17)	**204**

a 163 strains were evaluated for both drugs, plus 41 for Sb^V^ only, and 41 for HePC only yielding a total of 204 strains for each drug and 245 for one or both drugs.

### Ethics statement

This study was approved and monitored by the Ethics Committees of CIDEIM and CDFLLA for the use of *Leishmania* strains and clinical information of the corresponding patients in accordance with national and international guidelines for Good Clinical Practice. Prior written informed consent for the use of information from clinical histories had been obtained at the time of diagnosis from patients included in this study.

### Resistant and sensitive lines of Leishmania

Internal standards consisting of the sensitive strain (MHOM/COL/86/1166) and the experimentally derived Sb^V^ resistant line (MHOM/COL/86/1166-1000.1) [23] and HePC resistant line (MHOM/COL/86/1166-LUC056) [24] were included in each experiment to confirm the discriminatory capacity of the assays. The Sb^V^ and HePC resistant lines were propagated and maintained in the presence of 1000 µmoles Sb^III^/L and 60 µmoles HePC/L, respectively.

### Drugs

Additive-free meglumine antimoniate (Sb^V^; Walter Reed 214975AK; lot no. BLO918690-278-1A1W601; antimony analysis, 25%–26.5% by weight) and 1-hexadecylphosphocholine (HePC; miltefosine; Cayman Chemical Co., Ann Arbor, MI) were utilized for *in vitro* susceptibility evaluation. Trivalent antimony (SbIII) as potassium antimonyl tartrate thrihydrate was obtained from Sigma–Aldrich Chemical Company, St. Louis, MO.

### *In vitro* assay for drug susceptibility using intracellular amastigotes

Susceptibility was determined based on reduction of intracellular parasite burden in U-937 macrophages (ATCC CRL-159.3) as described by Fernández et al 2012 [24]. Briefly, 1.2×10^5^ U-937 cells were differentiated to macrophages by treatment with phorbol 12-myristate 13-acetate (PMA; 100 ng/ml; Sigma), then infected with promastigotes opsonized with 10% AB positive human serum at a ratio of 5 parasites per macrophage. Infected cells were incubated for 24 hours to allow differentiation of intracellular parasites to amastigotes. Afterwards supernatants were replaced with complete RPMI containing 16 µM HePC or 32 µg Sb^V^/ml. In the case of Sb^V^, medium containing 32 µg Sb^V^/ml was replenished 48 h later and incubation continued for an additional 24 h. HePC exposure was conducted over 48 h without replenishment [24].

Infection was assessed blindly by one of two experienced microscopists who evaluated all slides for this study. Four replicates of infected cells exposed to each drug and unexposed infected control macrophages were evaluated. The number of intracellular amastigotes per cell was determined for 100 macrophages per replica. Susceptibility was expressed as percent reduction of infection, determined by comparing parasite burden of infected cells exposed to the drug versus that of infected cells without drug.

The cutoff defining sensitive and resistant strains for each drug *in vitro* was based upon previously published analyses [24]. However, for the current study, an indeterminate range of parasite reduction was defined based on the absence of data within this range in the dataset used to derive the cutoff. Strains presenting a reduction of parasite burden between 35% and 48% for Sb^V^ and between 44% and 56% for HePC, were considered to have indeterminate susceptibility, Supplemental Figure S1. Therefore *in vitro* sensitivity or resistance was defined respectively by reduction of parasite burden above or below the indeterminate range for the corresponding drug.

### Genotyping of strains by multilocus microsatellites typing (MLMT)

In order to examine the relationship between genotypic diversity and drug-susceptibility profiles, the relative genetic diversity and proximity of *L. V. panamensis* strains pertaining to zymodemes 2.2 (n = 10) and 2.3 (n = 10), plus 12 other strains of this species without zymodeme classification from the Pacific coast region, and *L. V. guyanensis* strains from the Amazon (n = 9) and Andean region (n = 8) were analyzed by multilocus microsatellite typing (MLMT). The geographic distribution of *L. V. guyanensis* and its principal vector *Lutzomyia umbratilis* in Colombia, had previously been limited to the Amazon River basin. Therefore, in addition to drug susceptibility, the genetic diversity and relationship of the *L. V. guyanensis* populations involved in the 2003–2005 epidemic of domestic transmission by *Lutzomyia longiflucosa* in Chaparral, Tolima in the Andean foothills [25], [26] and strains of *L. V. guyanensis* from patients occupationally exposed to sylvatic transmission in the Amazon region were also analyzed. DNA was extracted from log-phase promastigotes using the Quigen Blood & Tissue Kit (Qiagen, USA). Fourteen microsatellites distributed in 13 *Leishmania* chromosomes were amplified by PCR, as previously described [27]. The size of the microsatellites was determined by mobility of the PCR products in 4.5% agarose gels. Genetic distances were estimated using MSA software and populations and neighbor joining trees were constructed using MEGA 5.

### Statistical methods

Strains of *L. Viannia* from each region were selected using a simple random sampling technique. To explore differences in susceptibility to Sb^V^ and HePC of *Leishmania Viannia* strains according to species and geographic regions, Kruskal-Wallis (K-W) tests and Dunn's post-test for multiple comparisons were performed. Differences in the susceptibility to Sb^V^ among *L. V. panamensis* strains from two endemic foci during two 10-year time periods, and between zymodeme 2.2 and 2.3 were analyzed using the Mann-Whitney non-parametric test. Association between *in vitro* susceptibility of *L. V. panamensis* to Sb^V^ and zymodeme, age or occupation was examined using contingency tables (Chi square). Comparison of proportions was calculated using the Z test. Correlation of susceptibility to HePC and Sb^V^ was evaluated based on the Spearman test. *P* values <0.05 were considered significant. Data were compiled in Microsoft Excel and analyzed using Prism 5 (GraphPad, Inc).

## Results

### Drug susceptibility of *Leishmania Viannia* species

**Figure 1:**
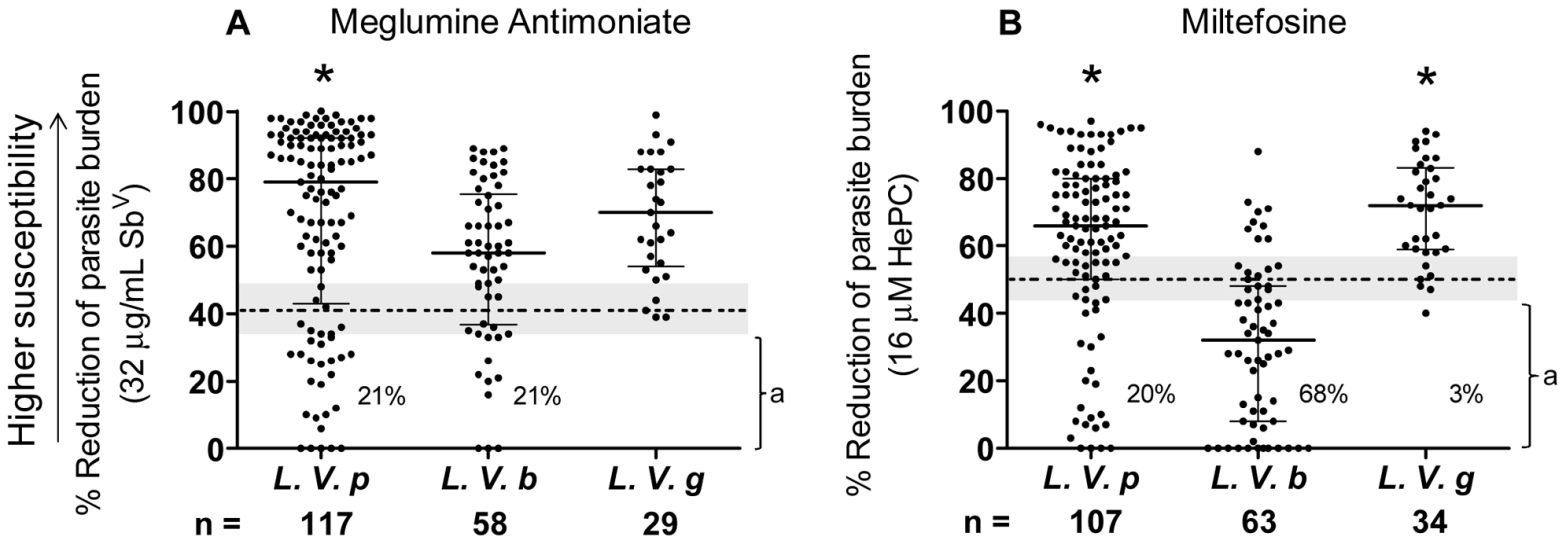
Susceptibility of *L. Viannia* species in Colombia to meglumine antimoniate and miltefosine. Reduction of parasite burden at the discriminatory concentration of anti-leishmanial drugs for clinical strains of *L. V. panamensis*, *L. V. braziliensis* and *L. V. guyanensis*. The cutoff thresholds (dotted horizontal line) of <41% reduction of parasite burden by Sb^V^ (A) and <50% reduction of parasite burden by HePC (B), and indeterminate zones (gray region) were defined based on previously described ROC curves as reduction of parasite burden between 35% and 48% for Sb^V^ and 44% and 56% for HePC (Fernandez O, et al 2012).^ a^ Strains presenting *in vitro* resistance. Kruskal-Wallis (K-W) for Sb^V^: *P* = 0.0009 and K-W for HePC: *P*<0.0001. * Dunn's multiple comparison test *P*<0.05: Compared with *L. V. braziliensis*.

Susceptibility to Sb^V^ and HePC differed among strains of the same species and between species of the (*Viannia*) subgenus (Figure 1). Quantitative analysis showed that *L. V. braziliensis* strains were significantly less susceptible to both drugs compared with other *(Viannia)* species. Qualitative analysis with respect to the cutoff thresholds and indeterminate zones of susceptibility *in vitro* revealed that *L. V. guyanensis* presented the highest proportion of sensitive strains for both drugs (82%, 28/34 for HePC and 86%, 25/29 for Sb^V^) with only one strain being classified as resistant to miltefosine and none for Sb^V^, whereas *L. V. braziliensis* exhibited the highest proportion of resistant strains (68% 43/63) for HePC (*P*<0.05), yet a high proportion (69%, 40/58) were sensitive to Sb^V^. The *in vitro* susceptibility of a comparatively small proportion overall of strains of these species of the *Viannia* subgenus fell within the indeterminate range: 8% for Sb^V^ and 16% for HePC.

Analysis of the distribution of susceptibility of strains of each species based on the median and inter-quartile range corroborated the high susceptibility of *L. V. panamensis* and *L. V. guyanensis* strains to Sb^V^ and HePC; over half of strains of these two species were highly sensitive, with ≥79% and ≥70% reduction of infection respectively, when exposed to 32 µg Sb^V^/ml, and ≥66% and ≥72% reduction respectively, when exposed to 16 µM HePC (6.5 µg/mL). In contrast, 50% of *L. V. braziliensis* strains presented *in vitro* resistance to the discriminatory concentration of HePC with ≤32% reduction of parasite burden. However, the inter-quartile distribution of the susceptibility of *L. V. braziliensis* strains to Sb^V^ revealed that 50% of *L. V. braziliensis* strains were moderately sensitive, with ≥58% reduction of parasite burden, and 25% were highly sensitive with ≥76% reduction of infection.

Only 10% (16/163) of strains evaluated *in vitro* for both drugs were resistant to both: 9% (7/80) *L. V. panamensis* and 17% (9/54) *L.V. braziliensis*. While correlation analysis of all strains using the Spearman test indicated a statistically significant relationship between the susceptibility to each drug (*P* = 0.0002), the attributable effect was very low, r = 0.283.

### Geographic variation in drug susceptibility

**Figure 2:**
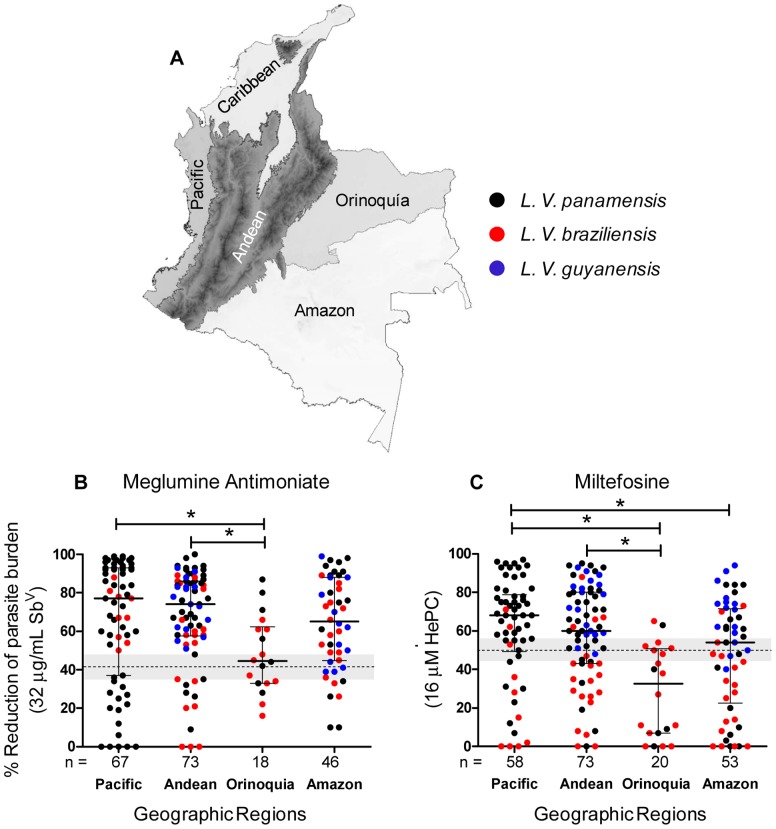
Susceptibility of *Leishmania Viannia* species from major natural geographic regions to meglumine antimoniate and miltefosine. Map of major natural geographic regions of Colombia (A). Susceptibility expressed as reduction of parasite burden at the discriminatory concentration of Sb^V^ (B) and HePC (C) in clinical strains of *L. V. panamensis, L. V. braziliensis* and *L. V. guyanensis.* Distributions of % reduction of parasite burden represent the median and the interquartile range. Qualitative classification of susceptibility is defined by the cutoff thresholds (dotted horizontal line) and indeterminate ranges are illustrated by gray shading. K-W for Sb^V^: *P* = 0.012 and K-W for HePC: *P*<0.0001. * Dunn's multiple comparison test *P*<0.05.

Exploratory analyses revealed geographic variation in drug susceptibility of the *L. Viannia* species most prevalent in different natural geographic regions. Strains from patients who acquired infection in regions east of the Andes mountain ranges (Orinoquía and Amazon regions) were significantly less susceptible to HePC than strains from the Pacific Coast region on the western side of the Andes, Figure 2. The lower susceptibility to HePC of strains from Amazon was influenced principally by *L. V. braziliensis* (K-W: *P* = 0.0003, Dunn's test: *P*<0.05). Strains originating from the Orinoquía region presented the lowest susceptibility to both drugs. Although most of the strains originating from this region were *L. V. braziliensis*, *L. V. panamensis* strains from this region also contributed to the lower susceptibility to HePC.

**Figure 3:**
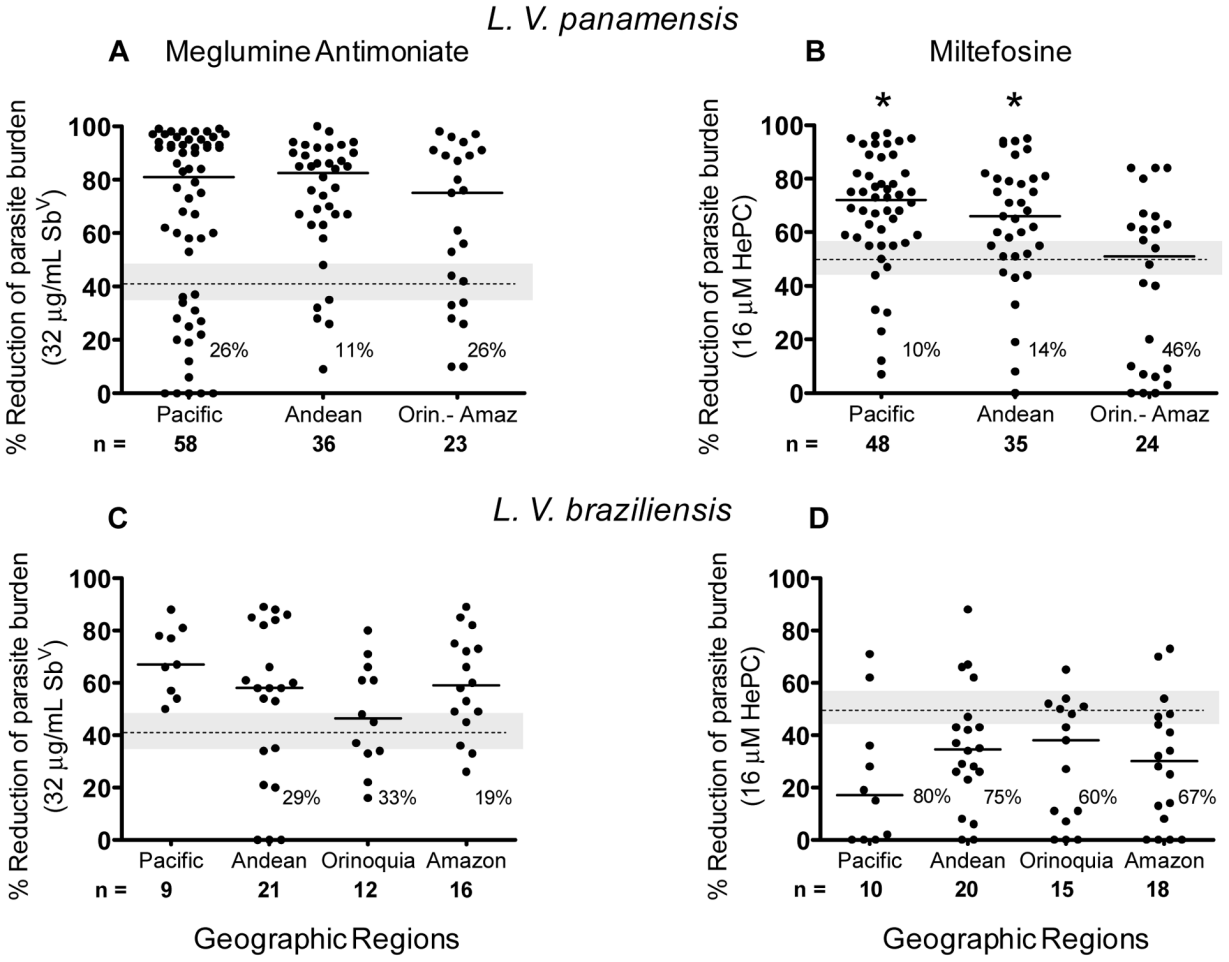
Susceptibility of *L. V. panamensis* and *L. V. braziliensis* to meglumine antimoniate and miltefosine by geographic region. Reduction of parasite burden at the discriminatory concentration of Sb^V^ (A and C) and HePC (B and D) for *L. V. panamensis* and *L. V. braziliensis* strains from Pacific, Andean, Orinoquía and Amazon regions. Because of the lower frequency of *L. V. panamensis* in regions east of the Andes mountain range, *L. V. panamensis* strains are combined for Orinoquía and Amazon regions. Qualitative classification of susceptibility is defined by the cutoff thresholds (dotted horizontal line) and indeterminate ranges are illustrated by gray shading. K-W for *L. V. panamensis* and HePC, *P* = 0.0012 and Dunn procedure, * *P*<0.05: Compared with Orinoquía and Amazon regions.

A significantly higher proportion of *L. V. panamensis* strains from the Orinoquía and Amazon regions presented *in vitro* resistance to HePC (46%, 11/24) compared with strains from Andean and Pacific regions (14%, 5/35 and 10%, 5/48 respectively); however, susceptibility of the same strains to Sb^V^ was similar across regions, Figure 3A and B. Notably, strains presenting resistance to miltefosine were isolated during and after 2005, and year of isolation was inversely correlated with intracellular parasite survival after exposure to 16 µM HePC (*P* = 0.043, r = −0.358) Supplemental Figure S2. Though not statistically significant, *L. V. braziliensis* strains from the Pacific region displayed greater susceptibility to Sb^V^ compared with other regions, but were less susceptible to HePC compared to strains of this species from other regions, Figure 3 C and D.

Strains presenting resistance to both HePC and Sb^V^ originated from the Andes, Orinoquia and Amazon regions. The frequency distribution by species of these *L. V. panamensis* and *L. V. braziliensis* strains displayed an inverse relationship from west to east of the Andes, Supplemental Figure S3.

### Susceptibility to antimony over time in Riverine foci of Tumaco

**Figure 4:**
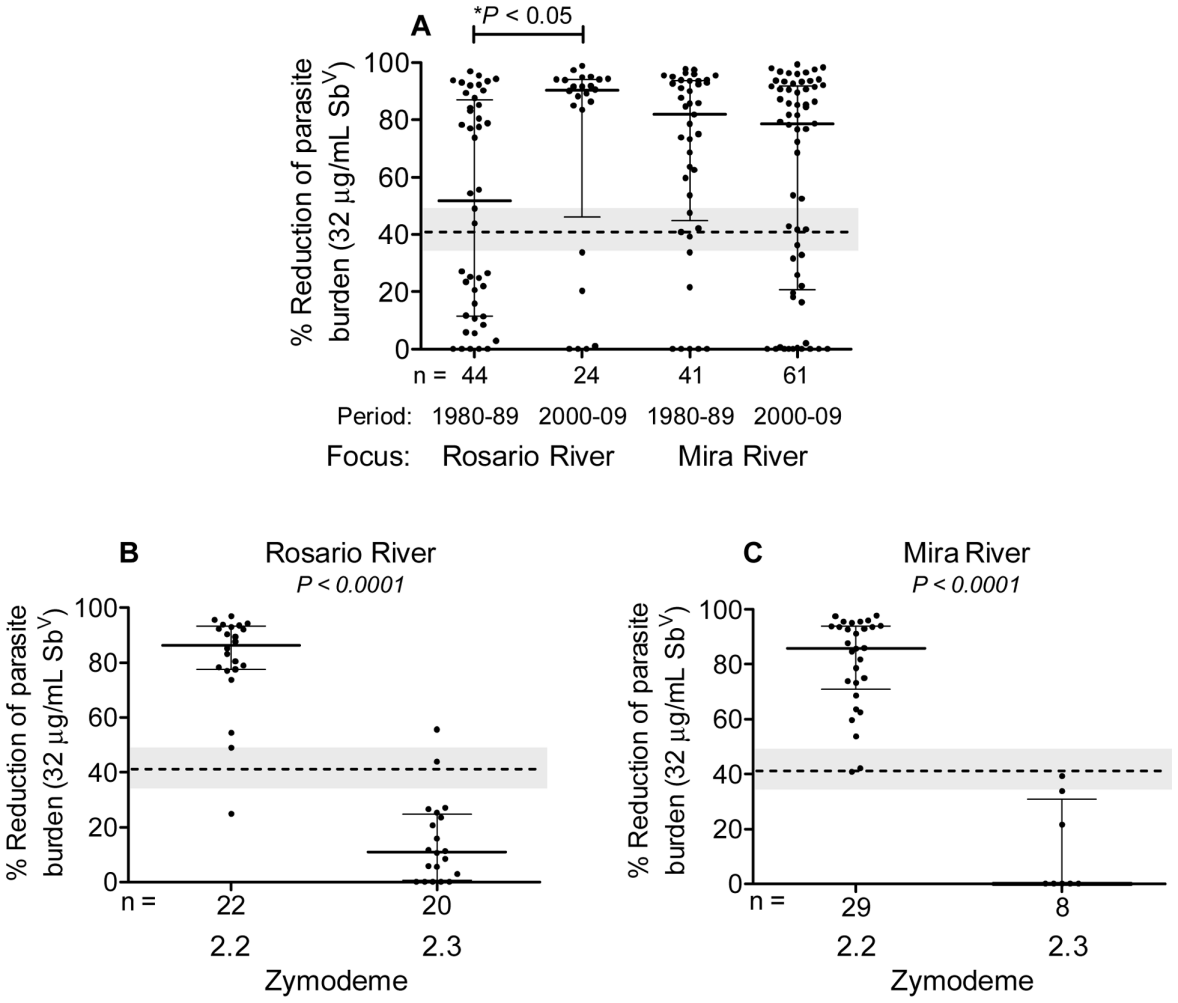
Susceptibility of *L. V. panamensis* strains to meglumine antimoniate in endemic foci of Tumaco and association of drug susceptibility with zymodeme. Distribution and median susceptibility of clinical strains from the Rosario River and Mira River foci during the decades 1980–1989 and 2000–2009. K-W, *P*<0.0001 and Dunn procedure (A). Comparison of susceptibility of strains pertaining to zymodeme 2.3 and zymodeme 2.2 isolated during 1980–1989 from the Rosario River and Mira River foci to Sb^V^ (B and C). Qualitative classification of susceptibility is defined by the cutoff thresholds (dotted horizontal line) and indeterminate ranges are illustrated by gray shading. Mann-Whitney, *P*<0.0001.

Evaluation of the susceptibility to Sb^V^ among *L. V. panamensis* strains (n = 170) from two endemic foci (Rosario and Mira Rivers) in the municipality of Tumaco, during two 10 year periods over 3 decades, revealed that strains from patients from the Rosario River focus presented a significantly lower median % reduction of parasite burden (*P*<0.05) and higher frequency of *in vitro* resistance to Sb^V^ during the decade 1980–89 (45%, 20/44) compared with the decade from 2000–09 (25%, 6/24). In contrast, the median % reduction of parasite burden was unchanged in the Mira River focus during the same periods but the frequency of resistance was significantly higher (*P*<0.05) during the period 2000–09 (38%, 23/61) compared with the earlier period 1980–89 (17%, 7/41), Figure 4A.

Surprisingly, discrete populations of *L. V. panamensis* were discernible at the upper and lower limits of susceptibility in the Rosario River focus during the period 1980–1989. Based on the median and inter-quartile range, a high reduction of parasite burden (>87%) was detected in 25% of strains and a very low (<11%) reduction of parasite burden was evident in 25% of strains from the Rosario River focus during the period 1980–1989. These populations continued to circulate during 2000–2009 in both foci but in different proportions, Figure 4A.

### Epidemiologic analysis of susceptibility to antimonials and isoenzyme polymorphism profile

Most *L. V. panamensis* strains isolated in the municipality of Tumaco during the decade 1980–89 had been previously classified by isoenzyme analysis as zymodeme 2.2 and 2.3, which corresponded to the most common isoenzyme profiles of *L. V. panamensis* in the southwestern region of Colombia [16]. Correlation of zymodeme with susceptibility to Sb^V^ revealed a significant association of the susceptibility phenotype with zymodeme (*P*<0.05) among strains isolated during the period 1980-1989, when zymodeme characterization was routine, Figure 4B and C. This association was confirmed in 20 strains isolated during the decade 2000–2009 (data not shown). Most resistant strains from both endemic foci pertained to zymodeme 2.3, whereas sensitive strains pertained to zymodeme 2.2. Furthermore, strains of zymodeme 2.3 were significantly less susceptible to Sb^V^ than strains belonging to zymodeme 2.2 (*P*<0.0001). Evaluation of these characteristics in strains pertaining to zymodemes 2.2 and 2.3 from other Departments within and outside of the Pacific coast region (n = 9), corroborated the Sb^V^ sensitive and resistant phenotype of these zymodemes that was observed in the Mira and Rosario River foci, (Data not shown).

**Figure 5:**
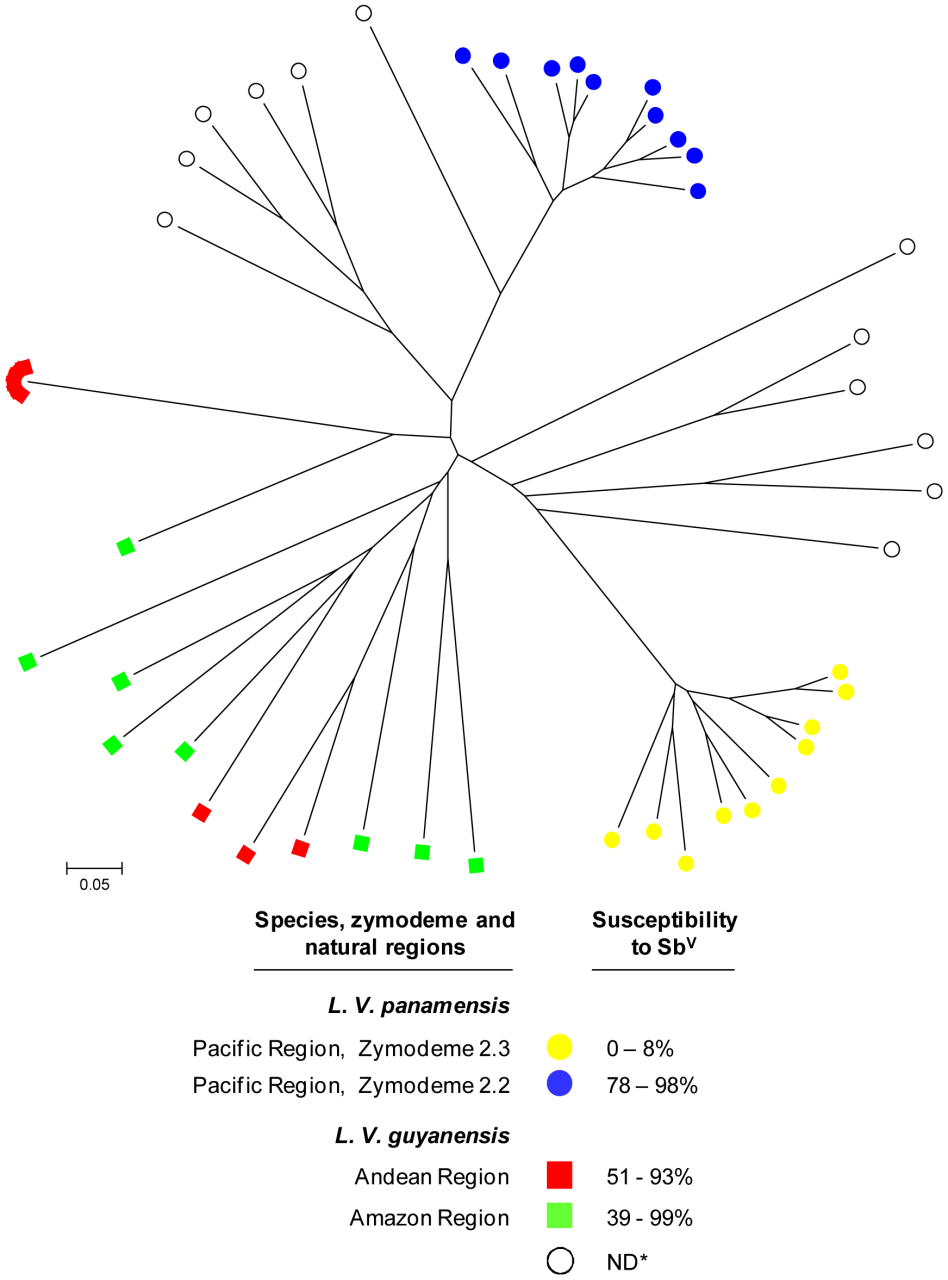
Analysis of genetic diversity of *L. V. panamensis* and *L. V. guyanensis* strains. The relationship between genetic diversity, zymodeme, drug susceptibility and geographic distribution was examined based on an unrooted neighbor joining tree generated from the distances calculated for microsatellite data. Strains included *L. V. panamensis* isolated from patients in the Municipality of Tumaco, Nariño pertaining to zymodemes 2.3 (n = 10) and 2.2 (n = 10), *L. V. panamensis* strains from the Pacific coast region not typed by isoenzyme analysis, and *L. V. guyanensis* strains isolated from patients from Andean (n = 9) and Amazon regions (n = 8). Range of susceptibility to antimony at 32 µg Sb^V^/ml is shown for the genetically discriminated groups of strains. ND* Zymodeme not determined.

Multilocus microsatellite typing (MLMT) revealed genetic diversity among the *L. V. panamensis* strains evaluated as well as two independent clusters that coincided with zymodeme 2.2 and zymodeme 2.3 and the susceptibility phenotype for antimonials, Figure 5. *L. V. guyanensis* strains were also genetically diverse within the two geographical regions represented; however, 6 of the 9 strains isolated in Chaparral, Tolima (Andean region) had identical MLMT profiles, suggesting the predominance of a clonal parasite population in this focus of transmission. No relationship between MLMT genotype and susceptibility to Sb^V^ was evident among the *L. V. guyanensis* strains, which displayed limited variability in drug susceptibility.

**Figure 6:**
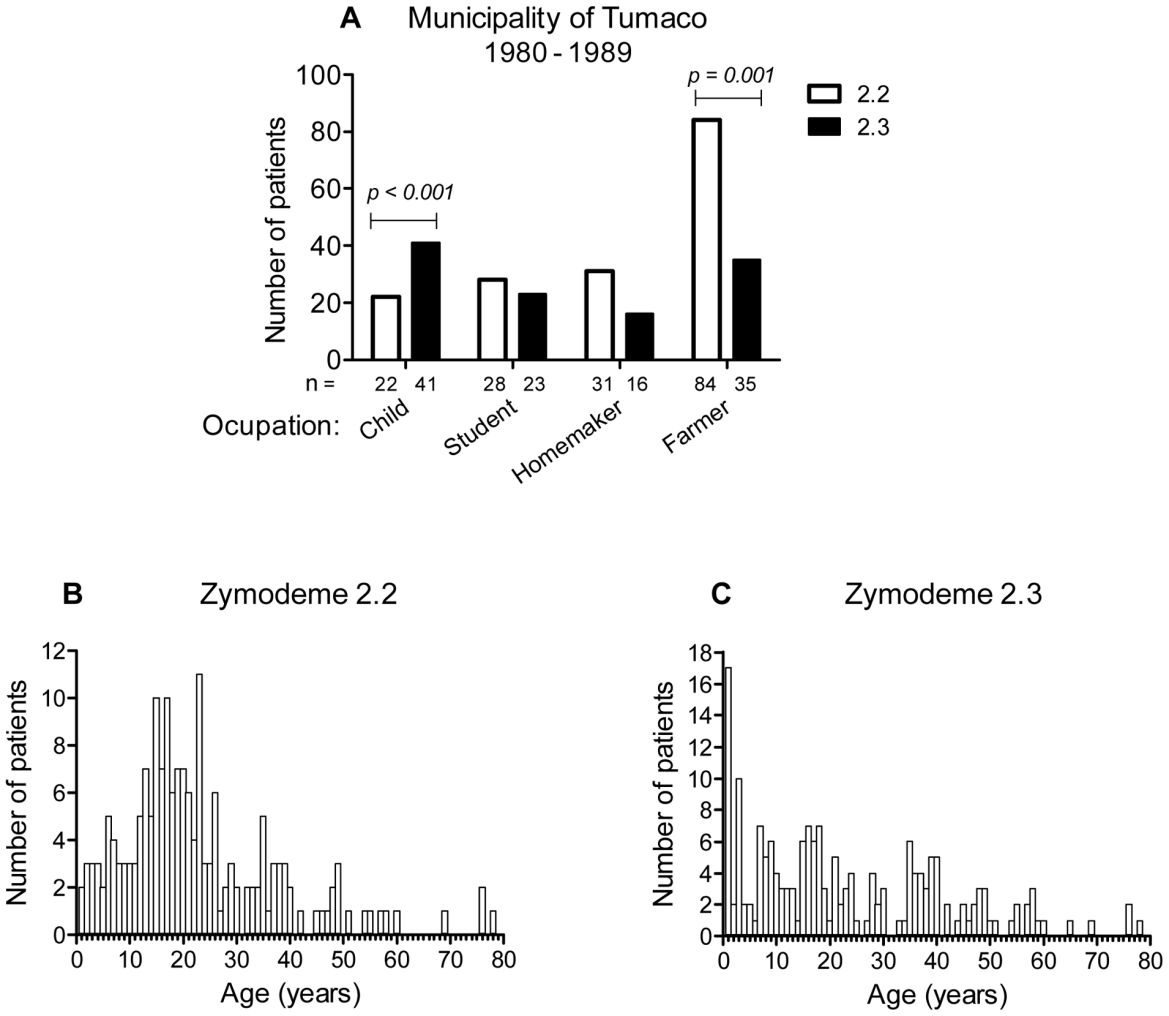
Association of zymodeme, age and occupation in the Municipality of Tumaco. Children <7 years of age were more frequently infected with strains of *L. V. panamensis* pertaining to zymodeme 2.3, while farmers were more often infected by strains belonging to zymodeme 2.2. n = 280 (A), Chi-square test, *P*<0.05. Distribution of *L. V. panamensis* strains belonging to zymodeme 2.2 (n = 181) and 2.3 (n = 149) by age of patients with cutaneous leishmaniasis from the Municipality of Tumaco, 1980–1989 (B and C).

Associations were also detected between zymodeme, patient age and occupation. A significantly higher number of strains isolated from children under seven years of age in the Municipality of Tumaco pertained to zymodeme 2.3, Figure 6A. In contrast, a significantly higher number of strains isolated from individuals involved in agricultural activity pertained to zymodeme 2.2. Frequency distributions of strains by zymodeme and according to the age of the corresponding patient yielded epidemiologic profiles characteristic of sylvatic transmission for zymodeme 2.2 and domestic transmission for zymodeme 2.3, Figure 6B and 6C.

## Discussion

This comprehensive evaluation of drug susceptibility of clinical strains of *Leishmania Viannia* species is novel in several aspects: the magnitude of the sample of clinical strains; the defined timeframes and geographical distribution represented by the strains included; the analyses of strains pertaining to three species of the *Viannia* subgenus; and the correlation of susceptibility to Sb^V^ with isoenzyme phenotype and demographic characteristics of the corresponding patients. The results of this study provide a detailed profile of susceptibility to meglumine antimoniate and miltefosine, the first and second line drugs currently used to treat dermal leishmaniasis in Colombia and most of Latin America, for the most prevalent species in regions of high transmission in Colombia.

The clinical response to antimonial drugs and miltefosine in different geographical contexts has long suggested differences in drug efficacy for disease caused by different species. In this *in vitro* analysis, strains of *L. V. guyanensis*, a species that has only recently been encountered in the context of domestic transmission in Colombia [25], were found to be consistently sensitive to both Sb^V^ and HePC. In contrast, 20% of strains of *L. V. panamensis*, the most prevalent species in Colombia [16], [17], [18] and frequently transmitted in the domestic setting, were resistant to Sb^V^ or HePC *in vitro*. Although a similar proportion of strains of *L. V. braziliensis* were resistant to Sb^V^, the reduction of parasite burden at the Sb^V^ concentration approximating Cmax in plasma during treatment was significantly lower for this species than for *L. V. panamensis*. Furthermore, overall *in vitro* susceptibility of *L. V. braziliensis* strains to miltefosine was remarkably low, with the majority (68%) being resistant to 16 µM (6.5 µg/mL) miltefosine, a concentration that distinguishes WT from experimentally selected lines [24]. This finding concurs with the lower clinical response to HePC reported in patients infected with *L. V. braziliensis* [4], [20] in some geographical areas [4] or occupational contexts [5].

In populations occupationally exposed to the sylvatic cycle of transmission of *L. V. braziliensis*, such as military personnel, and in regions where *L. V. braziliensis* is the predominant cause of dermal leishmaniasis, the risk of poor therapeutic outcome with HePC may be higher. Nevertheless, as for other antimicrobial agents, *in vitro* susceptibility does not necessarily predict individual clinical outcomes [28], [29], [30]. Rather, the risk or frequency of treatment failure increases in relation with the minimum inhibitory drug concentration (MIC) or IC_50_. However, clinical response, which is multi-factorial, can be achieved despite *in vitro* resistance to a given drug [31] as illustrated by the high proportion of dermal leishmaniasis patients infected with *L. V. braziliensis* in Colombia, Brazil and Bolivia that have responded to treatment with HePC [5], [7], [8]. Nevertheless, the high frequency of *in vitro* resistance to HePC among *L. V. braziliensis* strains circulating in Colombia underscores the importance of systematic follow-up of treatment and the rationale for combined therapies that could reduce the risk of failure and selection of resistant populations.

Clinical response data and pharmacokinetic considerations of drug exposure in designing and interpreting *in vitro* susceptibility are sorely needed to reliably characterize the relationship between clinical outcome and drug susceptibility. Recognizing that clinical response and *Leishmania* species are not routinely determined outside of clinical trials, this relationship would be most informatively addressed within the scope of randomized, controlled clinical trials in which many of the factors influencing clinical response are controlled.

*In vitro* evaluation of anti-leishmanial drug susceptibility of clinical strains relies on quantitative assessment of intracellular survival of amastigotes following exposure of infected host cells to the corresponding drugs [32], [33]. The recent implementation of an assay based on the burden of surviving intracellular parasites after exposure to a single drug concentration that discriminates WT and experimentally selected resistant populations [24] was critical to the feasibility of this large scale study. The results substantiated a wide spectrum of susceptibility within each species, illustrating the importance of evaluating a representative sample of strains that is inclusive of the diversity of the species.

The analysis of clinical strains from different geographic regions revealed lower susceptibility to both HePC and Sb^V^ among populations of *Leishmania* originating east of the Andes mountain range, an important natural geographic barrier. Although the proportions of the three species from patients originating from the eastern and western sides of the Andes differed, overall, *Leishmania* strains from the Pacific and Andean regions were more sensitive to both drugs than strains from Orinoquía and Amazon regions, Figure 2. The finding that *L. V. panamensis* strains isolated from patients originating in Orinoquía and the Amazon region were significantly less susceptible to HePC than strains of this species originating from the Andean and Pacific Coast regions, Figure 3, raises the question of whether populations of this species acquired by occupationally exposed individuals may have derived from a cycle of transmission involving intrinsically less susceptible parasite populations, or alternatively, might reflect exposure to HePC treatment. Supporting the plausibility of drug pressure contributing to this pattern, the majority (79%) of *L. V. panamensis* strains from Orinoquia and Amazonia derived from cases diagnosed after 2005, and HePC-resistant strains were isolated from 2005 onward (Supplemental Figure S2), several from uniformed service personnel. However, multiple factors are likely to have contributed to the relationship between year of isolation and resistance to HePC. Zymodeme or microsatelite analyses of these strains may reveal whether distinct populations of *L. V. panamensis* transmitted within the ecological and epidemiological circumstances in these regions contributed to the differences in susceptibility.

Examination of Sb^V^ susceptibility of *L. V. panamensis* strains isolated during the decade 1980–1989 from patients of two riverine foci in the municipality of Tumaco in relation with zymodeme analysis [16], showed that resistant strains corresponded with zymodeme 2.3 and susceptibilible to zymodeme 2.2. These phenotypic differences were also discernible at the genetic level; *L. V. panamensis* strains from both zymodemes independently clustered in the display of MLMT analyses. This finding and the confirmation of the susceptibility phenotypes of *L. V. panamensis* strains of zymodeme 2.2 and 2.3 from other areas of transmission within and outside of the Pacific coast region, provide compelling evidence of intrinsic differences in susceptibility within this species. Although unlikely to be directly involved in susceptibility to antimonials, isoenzyme polymorphisms and/or microsatellites associated with the susceptibility phenotype provide potentially exploitable markers for epidemiological applications and clinical decisions. These populations can also provide insight into the mechanisms involved in their divergent susceptibilities to antimony that may be relevant and useful across species.

Low drug susceptibility may be an intrinsic and/or acquired phenotype; a strain having low intrinsic susceptibility or tolerance could become further unresponsive as evidenced by some strains isolated pre-treatment with either Sb^V^ or HePC, and at treatment failure [12], [13]. Nevertheless, considering the long history of monotherapeutic use of pentavalent antimonials in Colombia and elsewhere in the region, evidence supporting the likelihood of anthroponotic as well as zoonotic transmission, and the documentation of acquired resistance in prospectively isolated clinical strains, the discrimination of intrinsic and/or acquired bases of drug susceptibility phenotype remains challenging. Intrinsic resistance to specific drugs resulting from absence of the corresponding molecular target as seen in particular phylogenic groups of bacteria has not been observed for any antileishmanial drug. Monotherapy could amplify intrinsic resistance, as well as promote acquired mechanisms. The biological cost or advantage of loss of susceptibility and the role of anthroponotic transmission will influence the impact of treatment policy and practice on the drug susceptibility of prevalent *Leishmania* populations.

The observed association of *Leishmania* zymodeme with patient occupation and age as well as parasite drug susceptibility in endemic riverine foci of the municipality of Tumaco supports the overlap of domestic and sylvatic transmission cycles among the inhabitants of these communities. The Sb^V^ resistance phenotype of zymodeme 2.3 and the association of this zymodeme with children in the communities of Tumaco suggest that domestic transmission may have been a factor in the emergence and dissemination of the zymodeme 2.3 population of *L. V. panamensis* in these foci during two decades spanning 30 years. The feasibility of assessing drug susceptibility using a single drug concentration and the precedent of being able to distinguish sensitive and resistant strains based on an intrinsic biochemical profile such as zymodeme provide opportunity to address this relationship. Meanwhile, the spectrum of susceptibility and frequency of *in vitro* resistance to first and second line treatments heighten the importance of alternative and combination treatment strategies.

## Supporting Information

Figure S1 Definition of discriminatory drug concentrations of meglumine antimoniate and miltefosine for susceptibility determination in *L. Viannia* species. The cutoff thresholds (dotted vertical line) of <41% reduction of parasite burden by Sb^V^ (A) and <50% reduction of parasite burden by HePC (B), and indeterminate zones (gray region) as reduction of parasite burden between 35% and 48% for Sb^V^ and 44% and 56% for HePC were defined based on previously described ROC curves (Fernandez O, et al 2012).
(TIF)

Figure S2 Correlation between year of isolation and miltefosine susceptibility of *L. V. panamensis* strains from Amazon/Orinoquia regions.
(TIF)

Figure S3 Geographic distribution by species, of strains presenting *in vitro* resistance to both meglumine antimoniate and miltefosine.
(TIF)
