# Correction: Miltefosine and Antimonial Drug Susceptibility of *Leishmania Viannia* Species and Populations in Regions of High Transmission in Colombia

**DOI:** 10.1371/journal.pntd.0003026

**Published:** 2014-06-20

**Authors:** The PLOS Neglected Tropical Diseases Staff

The latter surname of the 6^th^ author was omitted. The author’s full name is Isabel Rodríguez-Barraquer.
