# Supplementary figures and images for: Miltefosine and Antimonial Drug Susceptibility of Leishmania Viannia Species and Populations in Regions of High Transmission in Colombia
Source: PLoS Negl Trop Dis. 2014 May 22;8(5):e2871. doi: 10.1371/journal.pntd.0002871 (PMC4031164; doi:10.1371/journal.pntd.0002871)

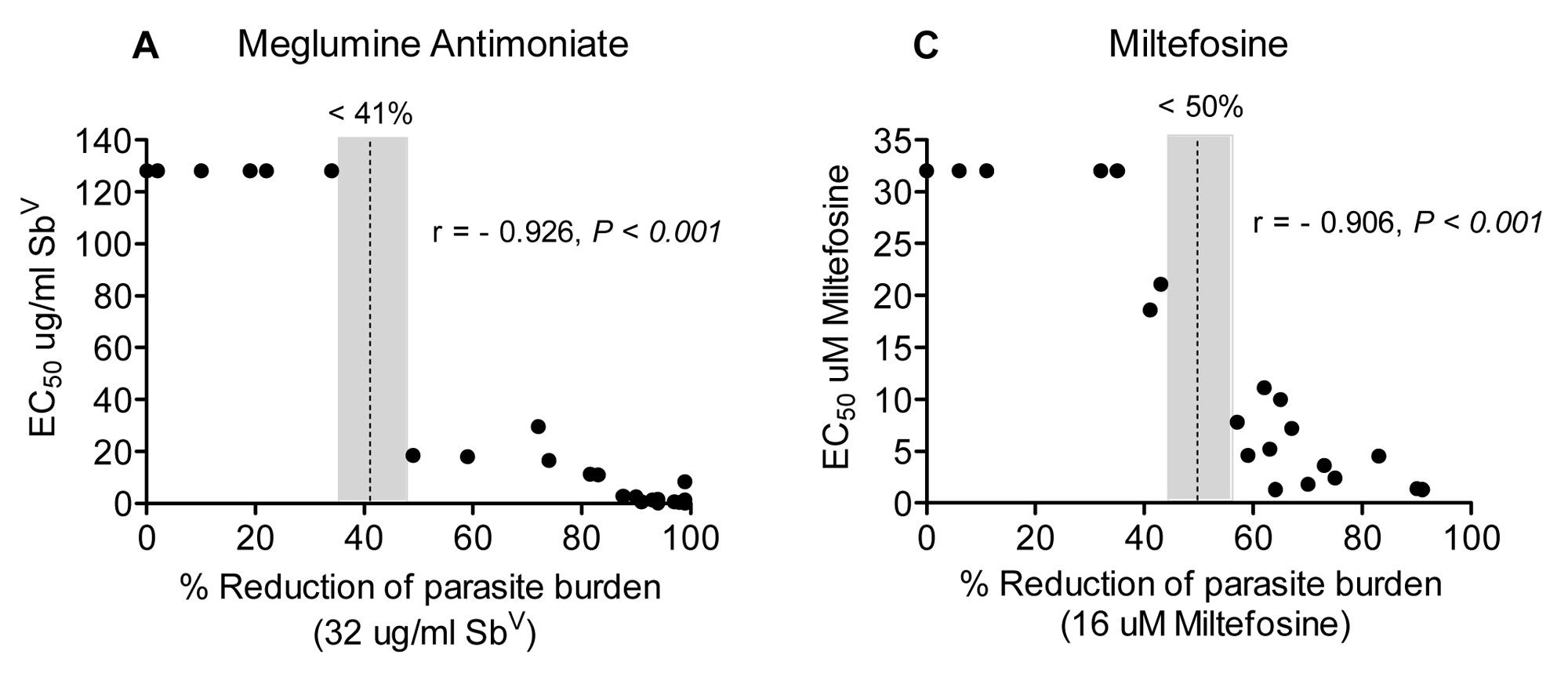

Supplement: Figure S1 — Definition of discriminatory drug concentrations of meglumine antimoniate and miltefosine for susceptibility determination in L. Viannia species. The cutoff thresholds (dotted vertical line) of <41% reduction of parasite burden by SbV (A) and <50% reduction of parasite burden by HePC (B), and indeterminate zones (gray region) as reduction of parasite burden between 35% and 48% for SbV and 44% and 56% for HePC were defined based on previously described ROC curves (Fernandez O, et al 2012). (TIF) [file pntd.0002871.s001.tif]

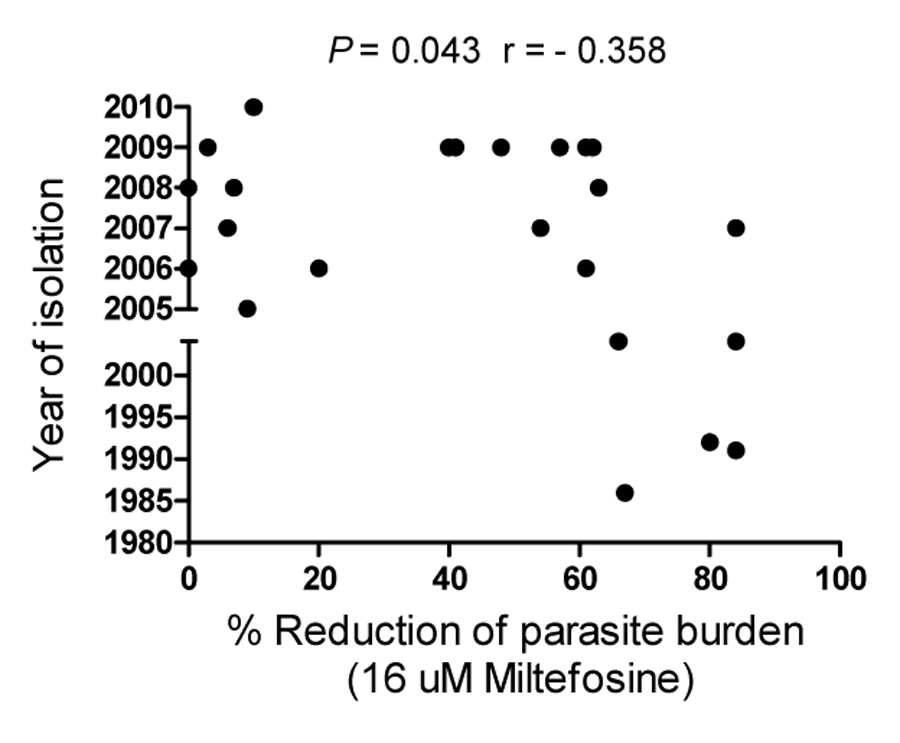

Supplement: Figure S2 — Correlation between year of isolation and miltefosine susceptibility of L. V. panamensis strains from Amazon/Orinoquia regions. (TIF) [file pntd.0002871.s002.tif]

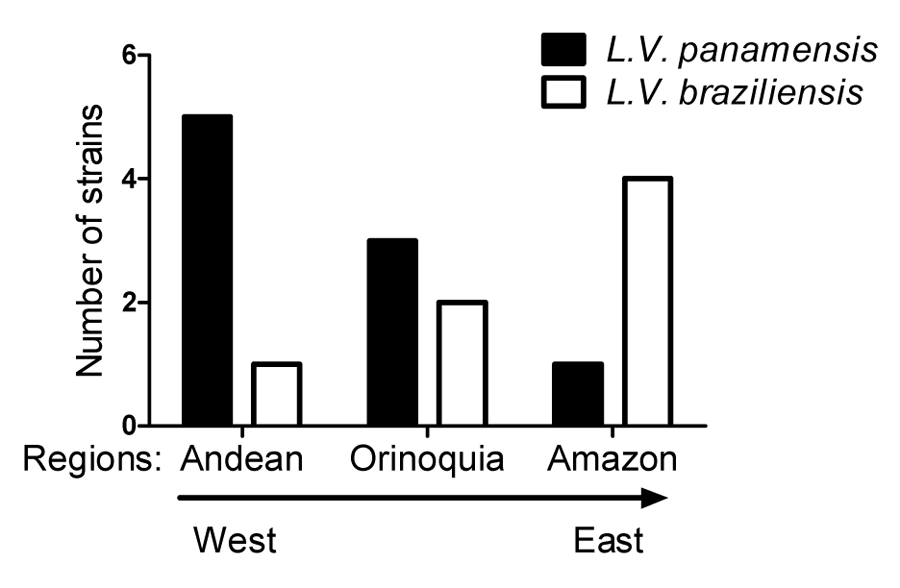

Supplement: Figure S3 — Geographic distribution by species, of strains presenting in vitro resistance to both meglumine antimoniate and miltefosine. (TIF) [file pntd.0002871.s003.tif]
